# Variations in the interaction of human defensins with *Escherichia coli*: Possible implications in bacterial killing

**DOI:** 10.1371/journal.pone.0175858

**Published:** 2017-04-19

**Authors:** Basil Mathew, Ramakrishnan Nagaraj

**Affiliations:** CSIR-Centre for Cellular and Molecular Biology, Hyderabad, India; Roskilde Universitet, DENMARK

## Abstract

Human α and β-defensins are cationic antimicrobial peptides characterized by three disulfide bonds with a triple stranded β-sheet motif. It is presumed that interaction with the bacterial cell surface and membrane permeabilization by defensins is an important step in the killing process. In this study, we have compared interactions of three human α-defensins HNP3, HNP4, HD5 and human β-defensins HBD1-4 that are active against *Escherichia coli*, with its cell surface and inner membrane as well as negatively charged model membranes. We have also included the inactive α-defensin HD6 in the study. Among the α-defensins, HNP4, HD5 and HD6 were more effective in increasing the zeta potential as compared to HNP3. Among the β-defensins, HBD1 was the least effective in increasing the zeta potential. The zeta potential modulation data indicate variations in the surface charge neutralizing ability of α- and β-defensins. Comparison of *E*. *coli* inner membrane and model membrane permeabilizing abilities indicated that HD5, HD6 and HBD1 do not permeabilize membranes. Although HBD4 does not permeabilize model membranes, considerable damage to the inner membrane of *E*. *coli* is observed. Our data indicate that mammalian defensins do not kill *E*. *coli* by a simple mechanism involving membrane permeabilization though their antibacterial potencies are very similar.

## Introduction

Human defensins are small cysteine rich cationic antimicrobial peptides with three disulfide bridges. Based on the disulfide connectivity, they have been classified into two major groups, α- and β-defensins [1]. The human genome contains five α-defensin genes, which codes for six α-defensins and approximately thirty β-defensin genes [2–6]. Human defensins show considerable variations in their amino acid sequences, except α-defensins HNP1-3 [7, 8]. The primary structures of HNP1-3 differ only by a single residue at the N-terminus [9]. The three dimensional structures of human α- and β-defensins are similar, consisting of a characteristic triple stranded antiparallel β-sheet structure connected by three disulfide bonds [10–15]. In the case of β-defensins, apart from the core β-sheet structure, a helix is also present at the N-terminal region [12–15]. A motif called “γ” core motif has been reported by Yount and Yeaman [16], which may have a role in modulating the activity of defensins [17]. Despite having similar structures, mammalian α- and β-defensins show considerable variations in their antibacterial potencies and spectrum of activity [18–23]. HNP1-3 are more active against certain strains of *Staphylococcus aureus* as compared to *Escherichia coli*, whereas HNP4 and HD5 show comparable activity against *E*. *coli* and *S*. *aureus* [18]. HD6 does not show antibacterial activity *in vitro* [18]. Human β-defensins also show variations in their activity. HBD1 and 2 are active predominantly against gram-negative bacteria, whereas HBD3 and 4 are active against gram-negative and gram-positive bacteria [19–24] In linear host defense peptides such as magainins [25, 26], cecropins [27, 28] and cathelicidins [29–31], their model membrane activity has been correlated to bacterial membrane permeabilization resulting in cell death. The interactions by α- and β-defensins with model membranes are highly variable [32–35] and their relevance to bacterial killing is not yet established unequivocally. Early investigations carried out with HNP1-3 showed sequential permeabilization of the outer and inner membranes of *E*. *coli* during killing of the bacteria [36]. Further, a membrane pore formation mechanism was proposed based on the crystal structure of HNP3 [11]. However, recent studies indicate that membrane activity of α-defensins may not necessarily correlate with bacterial killing [37]. The bactericidal mechanism of HNP1 against *Staphylococcus aureus* has been found to involve interaction with lipid II and inhibition of cell wall synthesis [37]. Human enteric α-defensins HD5 and HD6 do not permeabilize model membranes [38]. HD5 kills *E*. *coli* by localizing to the cytoplasm [38] and possibly interacting with DNA [39]. Unlike α-defensins, tertiary structures of human β-defensins (HBDs) do not favor mechanisms involving pore formation [12–14]. It appears that the electrostatic interaction between HBDs and bacterial membranes leads to destabilization of the membrane [40]. The exact mechanism by which human β-defensins permeabilize bacterial membranes is yet to be established unequivocally. Also, all human β-defensins do not form higher order oligomers in solution [12–14]. The activities of α- and β-defensins on model membranes or membranes of bacteria have not been compared in the same series of experiments. This would help direct comparison of their activities and also get better insights into the differences in their interaction with membranes. In this study, we compare membrane activities of four human α-defensins and four β-defensins against bacterial and model membranes. We also investigated whether human defensins form well defined aggregates by electron microscopy (EM). Primary structures of defensins used in this study are shown in Table 1. Our results indicate that contributions from membrane activity of human defensins to bacterial killing vary considerably.

**Table 1:** Primary structures of human defensins.

Defensin	Sequence^a^	Net Charge
HNP3	DC^**1**^YC^**2**^RIPAC^**3**^IAGERRYGTC^**2**^IYQGRLWAFC^**3**^C^**1**^	+2
HNP4	VC^**1**^SC^**2**^RLVFC^**3**^RRTELRVGNC^**2**^LIGGVSFTYC^**3**^C^**1**^TRV	+4
HD5	ATC^**1**^YC^**2**^RTGRC^**3**^ATRESLSGVC^**2**^EISGRLYRLC^**3**^C^**1**^R	+4
HD6	AFTC^**1**^HC^**2**^RRSC^**3**^YSTEYSYGTC^**2**^TVMGINHRFC^**3**^C^**1**^L	+2
HBD1	DHYNC^**1**^VSSGGQC^**2**^LYSAC^**3**^PIFTKIQGTC^**2**^YRGKAKC^**1**^C^**3**^K	+4
HBD2	GIGDPVTC^**1**^LKSGAIC^**2**^HPVFC^**3**^PRRYKQIGTC^**2**^GLPGTKC^**1**^C^**3**^KKP	+6
HBD3	GIINTLQKYYC^**1**^RVRGGRC^**2**^AVLSC^**3**^LPKEEQIGKC^**2**^STRGRKC^**1**^C^**3**^RRKK	+11
HBD4	ELDRIC^**1**^GYGTARC^**2**^RKKC^**3**^RSQEYRIGRC^**2**^PNTYAC^**1**^C^**3**^LRKWDESLLNRTKP	+7

^a^ Numbers in superscripts adjacent to cysteines denote disulfide connectivities.

## Materials and methods

### Materials

All human defensins used in this study were purchased from Peptides International, USA. N-(3-triethylammoniumpropyl)-4-(6-(4(diethylamino) phenyl-hexatrienyl)pyridinium dibromide) (FM4-64) and SYTOX green were obtained from Molecular Probes (Eugene, OR, US). Phospholipids POPC (1-palmitoyl-2-oleoyl-*sn*-glycero-3-phosphocholine) and POPG (1-palmitoyl-2-oleoyl-*sn*-glycero-3-phospho-(1-*rac*-glycerol)) (sodium salt) were from Avanti Polar Lipids (Alabaster, AL). Cholesterol and calcein were from Sigma-Aldrich. All the other chemicals used for this study were of the highest grade available.

### Antibacterial activity

Minimum bactericidal concentration (MBC) of human defensins against *E*. *coli* MG1655 was determined as described elsewhere [39, 41]. In brief, cells collected from the mid-log-phase were washed and resuspended in 10 mM sodium phosphate buffer (pH 7.4) containing 1% tryptic soy broth. The final cell density was adjusted to 10^6^ colony forming units (CFU)/mL. 100 μL of these cells were then incubated with varying concentration of defensins for 2 hours at 37°C. The cells were then spread onto nutrient-rich defensin free Luria-Bertani (LB) agar plates and incubated for 12–15 hrs at 37°C, colonies formed were counted and percentage of killing was calculated. The lowest concentration at which complete killing observed was taken as MBC.

### Zeta potential measurements

Zeta potential of the *E*. *coli* in the presence of human defensins was measured as described previously [39]. The concentration of peptide used was 5μM, except for HBD3 and HD5. In the cases of HBD3 and HD5, 0.5 μM and 2.5 μM were found to neutralize the *E*. *coli* surface charge almost completely. Therefore, in the cases of HBD3 and HD5, 0.5 μM and 2.5 μM of peptides were used, respectively.

### Time-lapse fluorescence confocal microscopy

Effect of defensins on the *E*. *coli* inner membrane was examined using time-lapse confocal fluorescence microscopy. The assay was carried out as follows. Bacteria from the mid-log-phase was collected and resuspended (final density was adjusted to 10^6^ CFU/mL) in 10 mM sodium phosphate buffer (pH 7.4). Cells were then treated with 3 μM FM4-64 for 20 minutes at room temperature to stain the inner membrane. FM4-64 is a lipophilic dye which stains the bacterial inner membrane [42]. Excess dye was removed by centrifuging. The FM4-64 stained cells were then treated with sub-lethal concentrations of defensins in presence of 5 nM of SYTOX green. The concentrations of the defensins used were; HBD1: 3 μM, HBD2: 3 μM, HBD3: 1 μM, HBD4: 3 μM, HNP3: 8 μM, HNP4: 4 μM, HD5: 2.5 μM, HD6: 5 μM. Cells were immediately transferred to chambered slides and images were recorded using 100X oil immersion objective on a Leica Ultraspectral microscope SP8 (Leica Microsystems). Argon 488 and HeNe 561 lasers were used to excite SYTOX green and FM4-64, respectively. Signals ranging from 510 to 560 nm and 600 to 740 nm were collected for SYTOX green and FM4-64, respectively. Images were processed using LAS-AFver3.1.3 (Leica Microsystems).

### SYTOX green uptake assay using fluorescent spectroscopy

Effect of defensins on the *E*. *coli* inner membrane was assessed by measuring the extent of intracellular accumulation of SYTOX green. Cells from mid-log-phase were collected, washed and resuspended in 10 mM phosphate buffer. The final density was adjusted to 5 × 10^6^ CFU/mL. Cells were then treated with 2 × MBC of defensins in presence of 200 nM SYTOX green. Enhancement in SYTOX green fluorescence, a direct measure of the extent of membrane permeabilization was monitored in a Flurolog 3–22 fluorescence spectrophotometer (Jobin Yvon, USA). Excitation and emission wavelengths used were 503 nm and 523 nm, respectively.

### Calcein release assay

Large unilamellar vesicles (LUVs) composed of POPC:POPG (1:1) containing 50 mM calcein were prepared by lipid extrusion method [43]. Desired amounts of lipids from their respective chloroform stocks were taken into a glass tube and dried under a nitrogen stream to form a thin uniform lipid film. The film was further dried under vacuum for 5 to 6 hours to remove trace amounts of organic solvents. After this step, the lipid film was hydrated with 10 mM sodium phosphate buffer (pH 7.4) containing 50 mM calcein at 4°C for 12 to 15 hours. Hydrated lipid films were then vortexed and passed through polycarbonate membrane having a pore diameter of 100 nm using a mini-extruder (Avanti Polar Lipid Inc.). Free calcein was removed by passing through a Sephadex G50 column. Calcein entrapped vesicles (25 μM) were then treated with increasing concentrations of defensins and extent of calcein released was measured in a Fluorolog 3–22 fluorescence spectrophotometer (Jobin Yvon, USA). Excitation and emission wavelengths used were 485 and 515 nm, respectively. 1% Triton X-100 was used for complete release of calcein.

### Transmission electron microscopy

Defensins were diluted to a concentration of 20 μM in 10 mM sodium phosphate buffer (pH 7.4) from their respective stocks solutions (dissolved in Milli-Q water). From these solutions, 5 μL was deposited on a carbon-coated Formvar 200-mesh copper grid. The grid was left undisturbed for about 5 minutes; excess buffer was then removed using Whatman filter paper. Grids were stained with uranyl acetate (2% w/v) for 45 seconds. Excess stain was removed and grids were used for recording the images. Images were recorded using JAM-2100 LaB6 transmission electron microscope (JEOL, Tokyo, Japan) at 100 kV.

## Results and discussion

### Comparison of antibacterial activity of human defensins against *E*. *coli*

In order to compare the antibacterial activity and bacterial membrane permeabilizing abilities of human defensins, we determined the MBCs against *E*. *coli* MG1655. The MBC values of human defensins against *E*. *coli* MG1655 are summarized in Table 2. The antibacterial potencies of human defensins against *E*. *coli* are of following order: HBD3>HD5>HBD4>HBD2 = HBD1>HNP4>HNP3>HD6.

**Table 2:** Minimum Bactericidal Concentration (MBC) of human defensins against *E*. *coli* MG1655.

Defensin	Minimum bactericidal concentration in μM^a^
HNP3	20
HNP4	10
HD5	2.5
HD6	10 (52.5)
HBD1^b^	8
HBD2^b^	8
HBD3^b^	2
HBD4^c^	5

^a^ Values given in the parentheses represent the maximum percentage of killing observed at the given concentration.

^b^ Values were taken from Krishnakumari *et al*. 2013[44].

^c^ Value was taken from Sharma and Nagaraj 2012 [45].

### Electrostatic interaction of human defensins with the *E*. *coli* surface

Cationic antimicrobial peptides (CAMPs) initially interact with bacterial cell wall components, followed by subsequent interactions with bacterial inner membrane and other intracellular components [46]. It has been proposed that initial electrostatic interaction with bacterial cell wall components is critical in determining the efficacy of CAMPs, as the abrogation of these interactions leads to attenuation of antibacterial activity [47–49]. In fact, Alves *et al*., have reported a correlation between extent of surface charge neutralizing ability and minimum inhibitory concentrations for amphipathic CAMPs [50]. We have previously shown that effective interaction of human defensin analogs with bacterial surfaces enhances antibacterial potency [39, 51, 52]. With a view to understand, how human defensins vary in their ability to interact with bacterial surfaces, we compared the extent of electrostatic interactions between the *E*. *coli* cell envelope and human defensins by measuring changes in the zeta potential of the *E*. *coli*.

**Fig 1:**
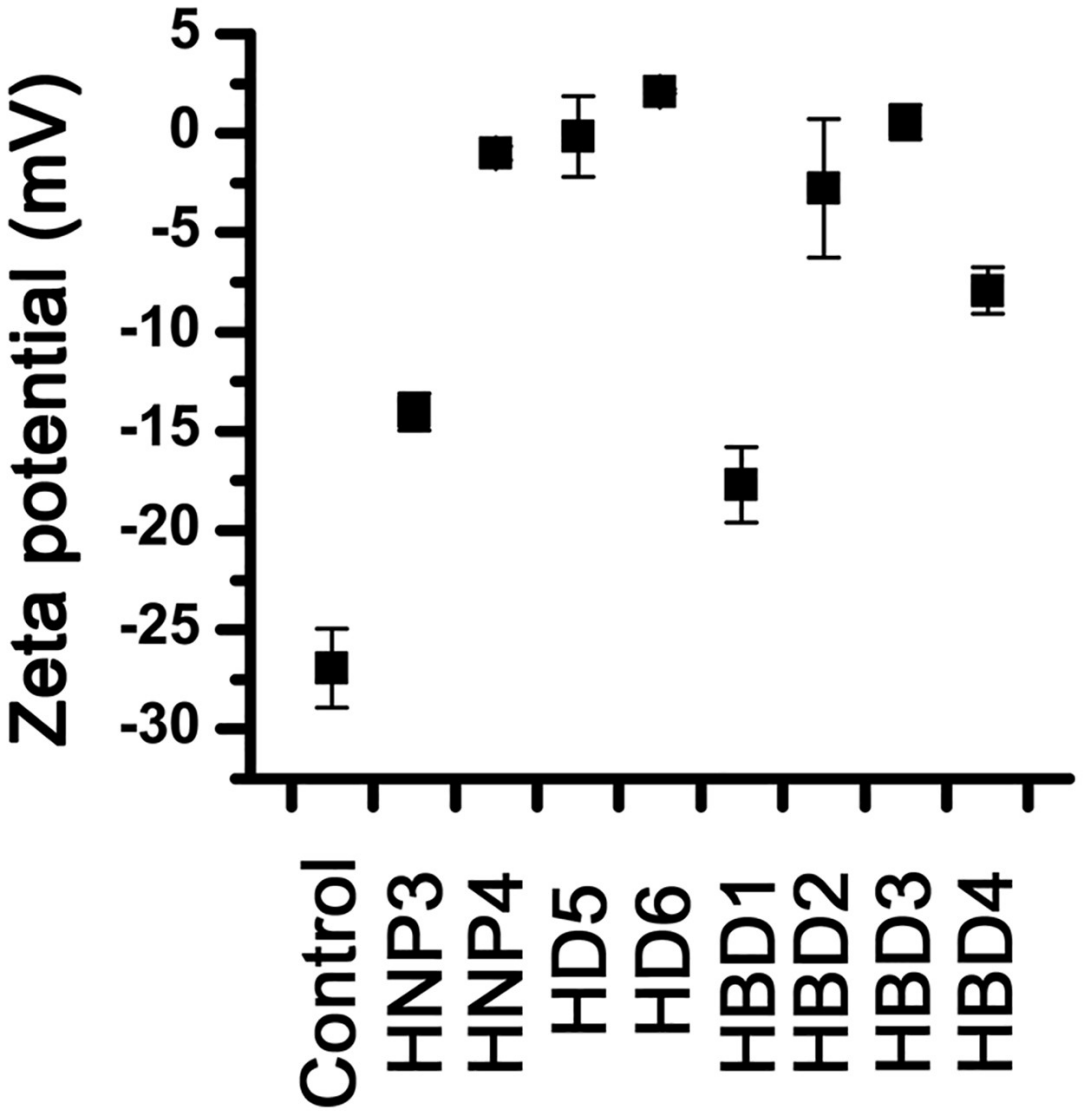
Effect of human defensins on the zeta potential of *E*. *coli*. Cells were treated with 5 μM defensin and zeta potential was measured, except in the cases of HD5 and HBD3. In the cases of HD5 and HBD3, zeta potential was measured after treating with 2.5 μM and 0.5 μM of peptides, respectively. Control represent zeta potential of *E*. *coli* in the absence of any peptide. Error bars represents standard deviations of three independent experiments.

Changes in the zeta potential of the *E*. *coli* in the presence of human defensins are shown in Fig 1. Among the defensins tested, HBD3 exhibits the most efficient bacterial surface charge neutralizing ability, which is presumably due to its very high net positive charge (+11). HNP3 and HBD1 show poor surface charge neutralizing ability as compared to other human defensins. Intriguingly, HD6, which has the lowest net positive charge (+2) among the human defensins tested, neutralizes the *E*. *coli* surface charge more efficiently than HBD4 (+7), HBD1 (+ 4) and HNP3 (+2). Also, HD5 which has same net positive charge (+4) as HNP4, neutralizes the *E*. *coli* surface charge at much lower concentration than HNP4. Clearly, the ability of human defensins to interact with bacterial surfaces is not merely governed by net positive charge alone. The inactive defensin HD6, neutralizes bacterial surface charge more effectively as compared to defensins that possess potent bactericidal activity. This could arise due to rapid oligomerization of HD6 on bacterial surfaces [53], resulting in effective charge neutralization. Analysis of tertiary structures of human defensins indicate that there are differences in the arrangement of cationic residues in three dimensional structures [10]. The observed variations in the ability of human defensins to associate with bacterial surfaces *via* electrostatic interactions could arise due to differences in the distributions of cationic side chains in the three dimensional structures of defensins.

### Effect of human defensins on the *E*. *coli* inner membrane

**Fig 2:**
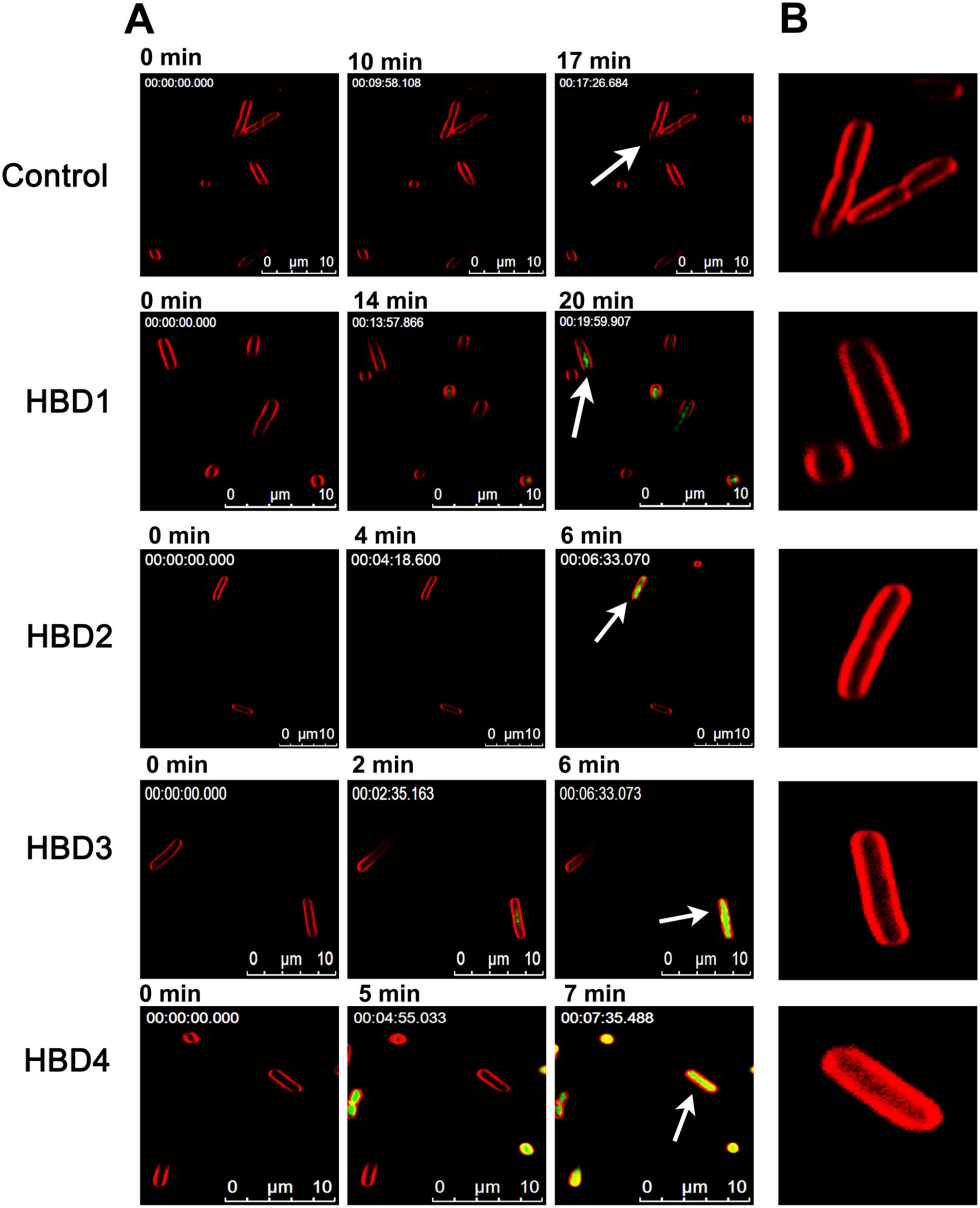
Effect of human β-defensins on the *E*. *coli* inner membrane. (A), Intracellular accumulation of SYTOX green in defensin treated cells as function of time. The minute at which images were recorded is mentioned above the respective image. Numbers given in the upper left corner of the images represent the elapsed time (h:min:s:ms). (B), Morphological features of FM4-64 stained *E*. *coli* inner membrane of selected bacteria, as indicated by arrows.

HNP1-3 kill *E*. *coli* by permeabilizing the outer and inner membranes in a sequential manner [36]. We next examined the effect human defensins on the *E*. *coli* inner membrane by monitoring intracellular accumulation of SYTOX green in defensin-treated cells using time-lapse fluorescence confocal microscopy. Confocal micrographs shown in Fig 2A indicate that not all human β-defensins permeabilize the *E*. *coli* inner membrane to the same extent. Intense accumulation of SYTOX green is observed only in the cases of HBD2-4. In the case of HBD1, faint accumulation of SYTOX green is evident at ~20min. However, the intensity is considerably low as compared to other membrane active defensins, suggestive of less extensive damage caused by HBD1 on the *E*. *coli* inner membrane. Intriguingly, both HBD1 and HBD2 show similar antibacterial efficacy against *E*. *coli* (Table 2). Therefore, the mechanism by which HBD1 kills *E*. *coli* is markedly different from HBD2. It has been proposed that bacterial killing mechanism of linear HBD1 is likely to involve interactions with cytoplasmic components [54]. Presumably, bacterial killing mechanism of HBD1 also follows similar pathways.

**Fig 3:**
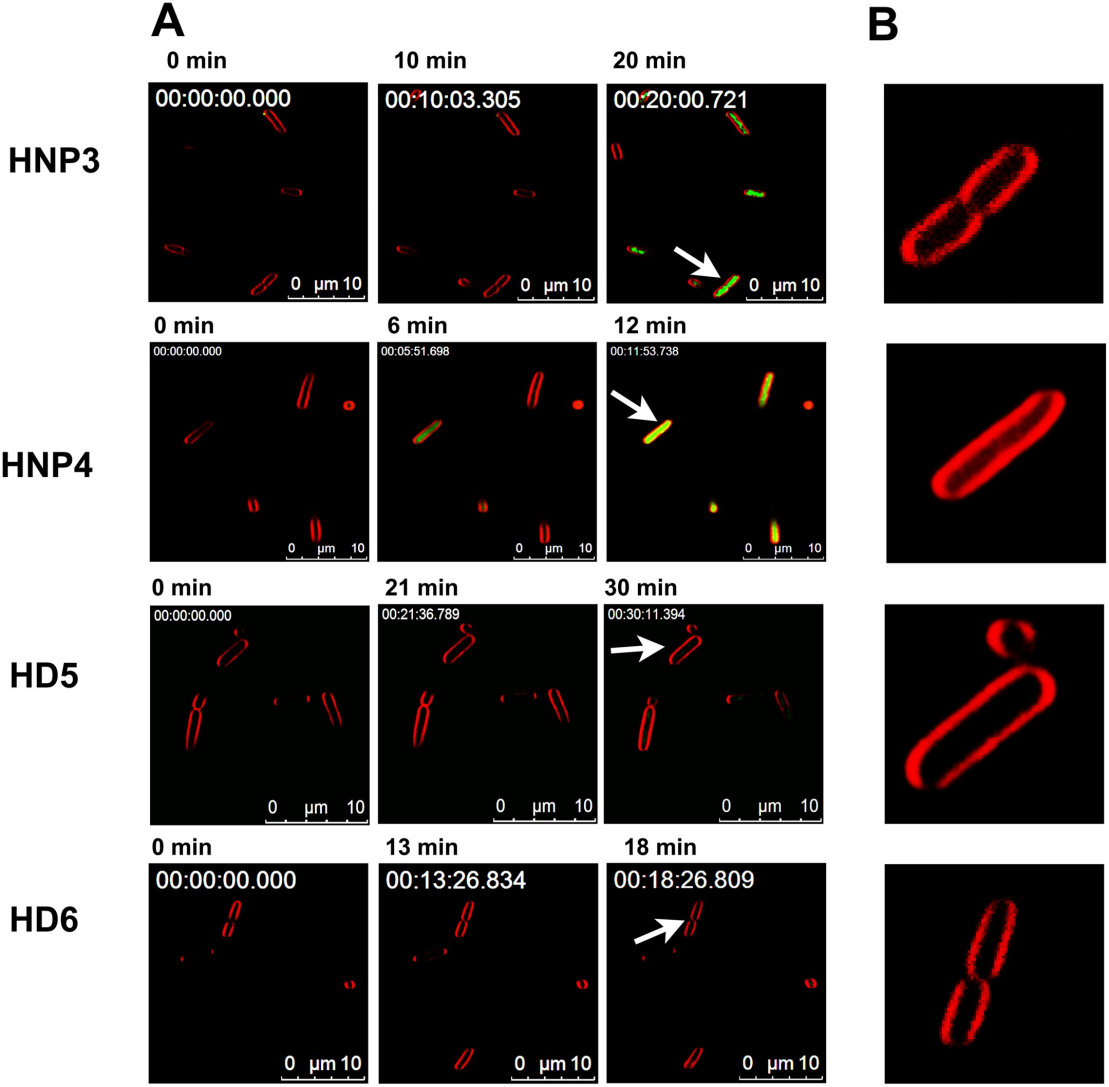
Effect of human α-defensins on the *E*. *coli* inner membrane. (A), Intracellular accumulation of SYTOX green in defensin treated cells as function of time. The minute at which images were recorded are mentioned above respective image. Numbers given in the upper left corner of the images represent the elapsed time (h:min:s:ms). (B), Morphological features of FM4-64 stained *E*. *coli* inner membrane of selected bacteria, as indicated by arrows. Numbers given in the upper left corner represent the elapsed time (h:min:s:ms).

In the case of α-defensins, cytoplasmic accumulation of SYTOX green is evident only in cell treated with myeloid defensins HNP3 and HNP4 (Fig 3A). Though enteric defensin HD5 kills bacteria more efficiently than HNP3 and HNP4, HD5 does not permeabilize the *E*. *coli* inner membrane. Recent findings indicate HD5 exerts its activity against the *E*. *coli* by localizing to bacterial cytoplasm [38] and possibly interacting with DNA [39]. In a very recent study, Wang *et al*., have shown that HD5 permeabilizes the *E*. *coli* inner membrane [55]. However, prolonged incubation (~40 minutes) was required to observe membrane perturbation [55]. Interestingly, analysis of time-lapse microscopy data indicates that, although extensive damage does not occur, a faint localization of SYTOX green occurs in *E*. *coli* treated with HD5 at 30 min (S1 Fig). It has been reported that degradation or topological changes in the bacterial DNA can a ffect the SYTOX green fluorescence [56]. Considering the fact that HD5 possess strong affinity towards DNA [39], its interaction with DNA could influence the fluorescence of SYTOX green, which may possibly account for the variations in the observed and reported inner membrane permeabilizing ability. However, both the results unambiguously suggest that perturbation occurs only after prolonged incubation with HD5. Undoubtedly, HD5 kills *E*. *coli* without causing extensive damage to bacterial membrane.

**Fig 4:**
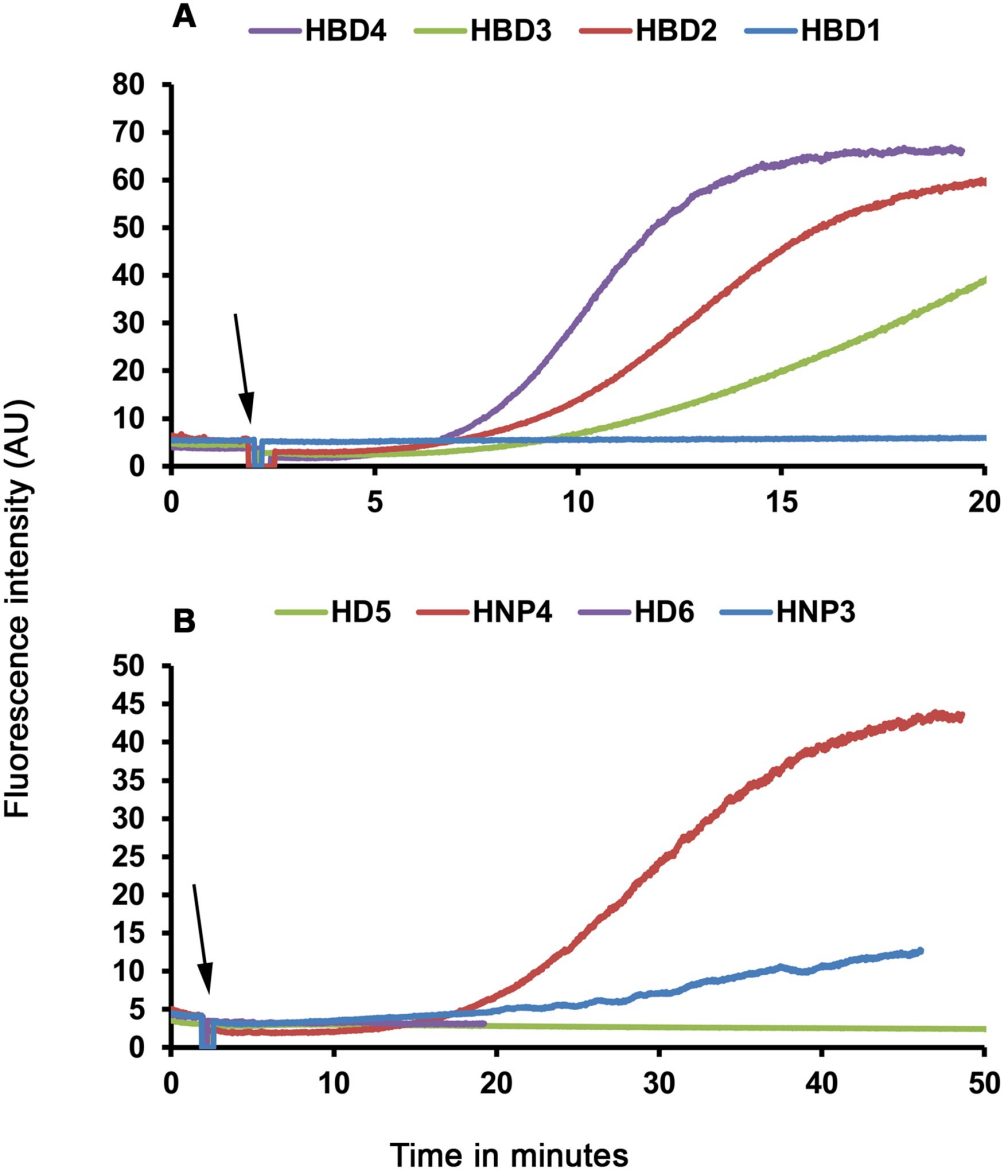
Accumulation of SYTOX green in human defensins treated *E*. *coli*. Cells were treated with 2 × MBC of (A), human β-defensins or (B), human α-defensins and intracellular accumulation of SYTOX green was monitored. The arrows indicate the point at which defensins were added.

Analysis of kinetics of SYTOX green accumulation indicate that membrane active β-defensins cause more rapid influx of dye into the bacterial cytoplasm as compared to membrane active α-defensins. In the case of HBD2-4, intense accumulation is evident in less than 7 min while in the HNP4 and HNP3 treated cells, accumulation is evident only at 11 min and 20 min, respectively. Further, we also observe that morphological features of the FM4-64 stained *E*. *coli* membranes (Figs 2B and 3B) are largely intact, except for HBD4 treated cells. In the case of HBD4 treated cells, FM4-64 staining shows considerable diffusion into cytoplasm, suggestive of more extensive damage caused by HBD4 as compared to other defensins. To further validate the observed variations in the bacterial membrane permeabilizing abilities, we examined intracellular accumulation of SYTOX green in the *E*. *coli* treated with 2×MBC of defensins using fluorescent spectroscopy. Results shown in Fig 4 further confirm time-lapse microscopy results. Clearly, human α-defensins HD5, HD6, and human β-defensin HBD1 do not cause extensive damage to the *E*. *coli* inner membrane. Together, time-lapse microscopy and fluorescent spectroscopy experiments indicate that human defensins do not permeabilize the *E*. *coli* inner membrane to the same extent although their antibacterial potencies are similar. Thus, there appears to be no unifying mechanism of bacterial killing, particularly against *E*. *coli*.

The evolutionary and functional relevance of primary structures of human defensins have been a subject of extensive investigations [57, 58]. In an attempt to understand the evolutionary relationships between α-defensin genes, Das *et al*., classified primate α-defensins into three different phylogenitic classes, class I, II and III [58]. According to this classification, HD5 belongs to class I, HD6 belongs to class II and HNP1-4 belong to class III. Interestingly, based on the structural analysis, Das *et al*., even predicted that variations in the electrostatic surface distributions could lead to differences in their bacterial killing mechanisms [58]. Our observations on the bacterial membrane permeabilizing abilities of human defensins strongly support this hypothesis. Also, it appears that amino acid selection is favored by environmental niches, as the enteric defensins HD5 and HD6 are non-lytic in nature. Although such hypothesizes are absent for human β-defensins, it is presumable that there too evolutionary selection have played pivotal role in rendering heterogeneity to bacterial killing mechanisms. Clearly, the poorly conserved amino acids play a critical role in modulating the bacterial killing mechanism.

### Interaction with lipid vesicles

**Fig 5:**
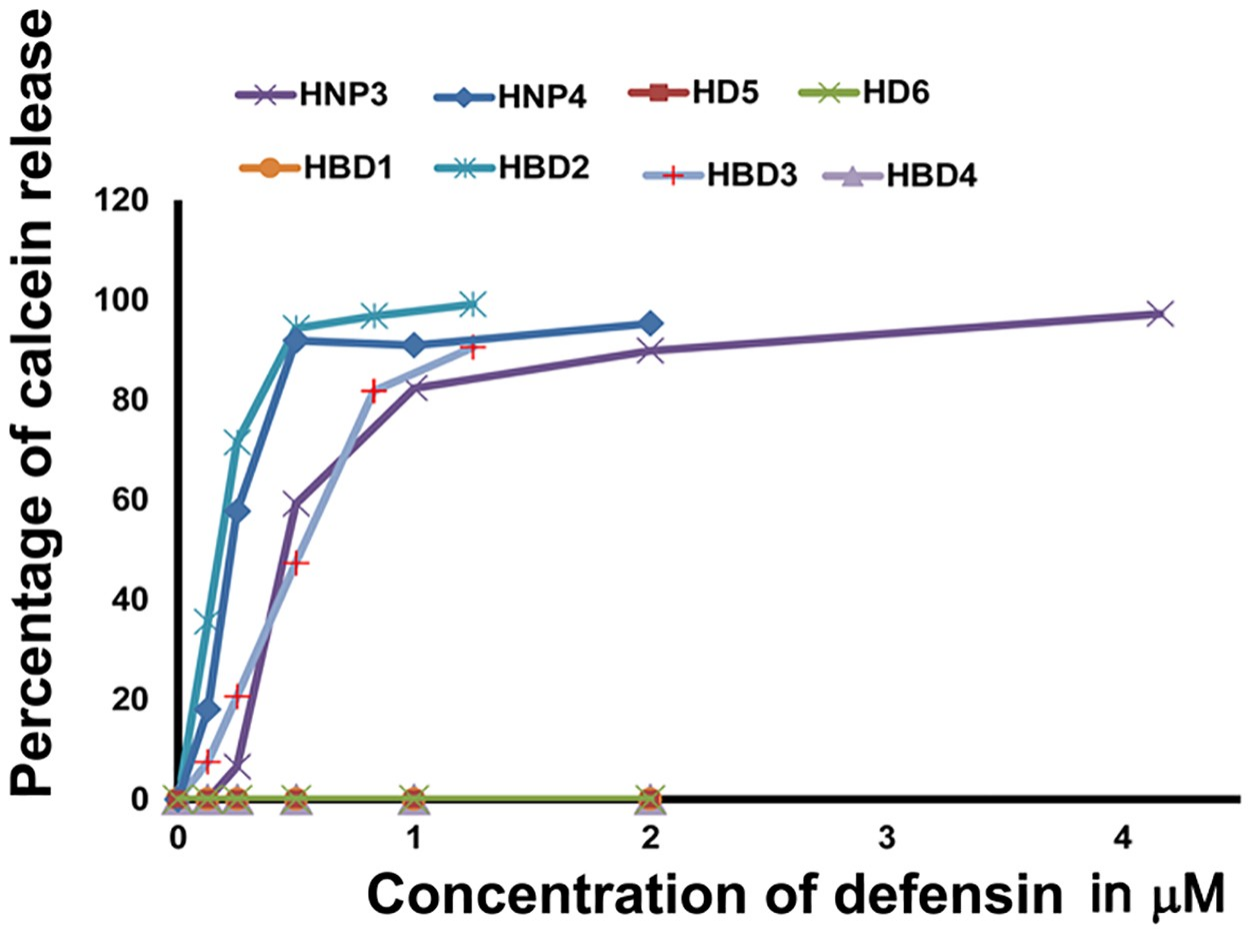
Effect of human defensins on POPC:POPG (1:1) vesicles. Calcein entrapped POPC: POPG vesicles (25 μM) were treated with increasing concentrations of human defensins and percentage of calcein released was calculated. Calcein released by 1% Triton X-100 was taken as 100%.

**Fig 6:**
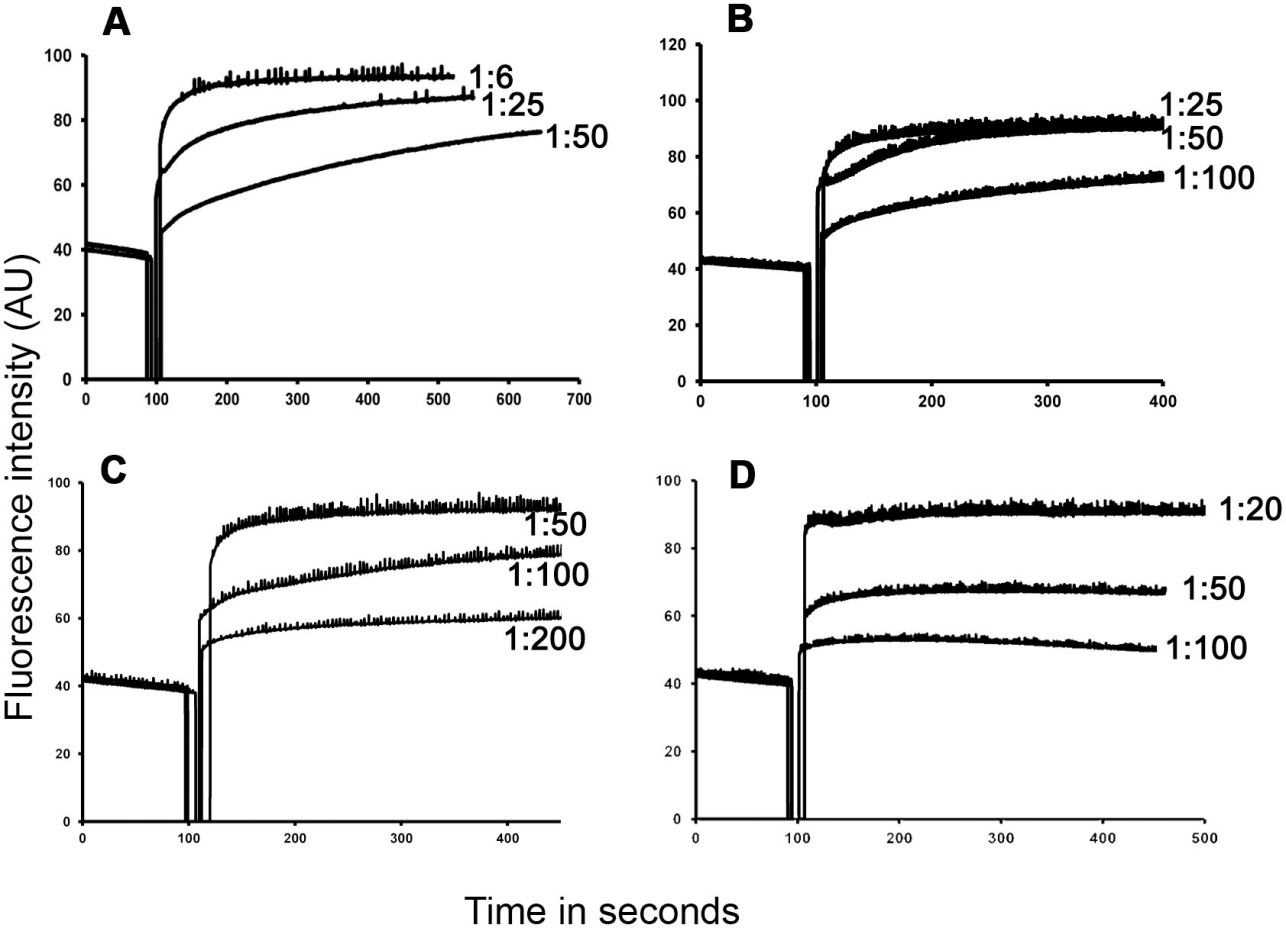
Kinetics of calcein release from POPC:POPG (1:1) vesicles treated with defensins. (A), HNP3; (B), HNP4; (C), HBD2 and (D), HBD3. The peptide to lipid ratios (P:L) are mentioned along the respective spectra.

Model membranes have been used as a tool to study mechanisms by which defensins permeabilize microbial membranes. There have been reports, which suggest that defensins permeabilize model membranes and cause their destabilization [32–35,59, 60]. We have examined the effect of α- and β-defensins on calcein entrapped negatively charged vesicles composed of POPC:POPG (1:1). We observe that with the exception of HBD1, HBD4, HD5 and HD6, defensins permeabilize lipid vesicles (Fig 5), which correlates with their ability to permeabilize the *E*. *coli* inner membrane, except for HBD4. It is evident that even low concentrations of defensins can affect the release of calcein. Further, analysis of kinetics of calcein release indicate that defensins cause a rapid release of calcein, although there are variations in the peptide to lipid (P:L) ratio where the maximum release is observed (Fig 6).

Based on the crystal structure and biophysical studies carried out with HNP1-3, it has been proposed that amphipathic dimer formed by human α-defensins form pores on bacterial membrane [11, 61]. Human enteric α-defensins, HD5 and HD6 also form amphipathic dimers similar to myeloid defensins [10]. However, they do not permeabilize model membranes [37]. Interestingly, detailed analysis of crystal structures indicated that amphipathic dimer formed by human enteric α-defensins are asymmetric in nature unlike HNP1-4 [10]. It is conceivable that the poorly conserved amino acids play a critical role in the observed variations in the dimer topology. Consequently, their ability to permeabilize lipid vesicles. Intriguingly, HBD4 which causes extensive damage to the *E*. *coli* inner membrane does not permeabilize PC:PG vesicles. The underlying physico-chemical reason behind this discrepancy is not clear at this point. It is possible that essential factors present on the microbial surface or membranes may be playing a critical role in determining the membrane activity of HBD4, as reported for other defensins [33, 62].

### Human defensins form ordered aggregates

**Fig 7:**
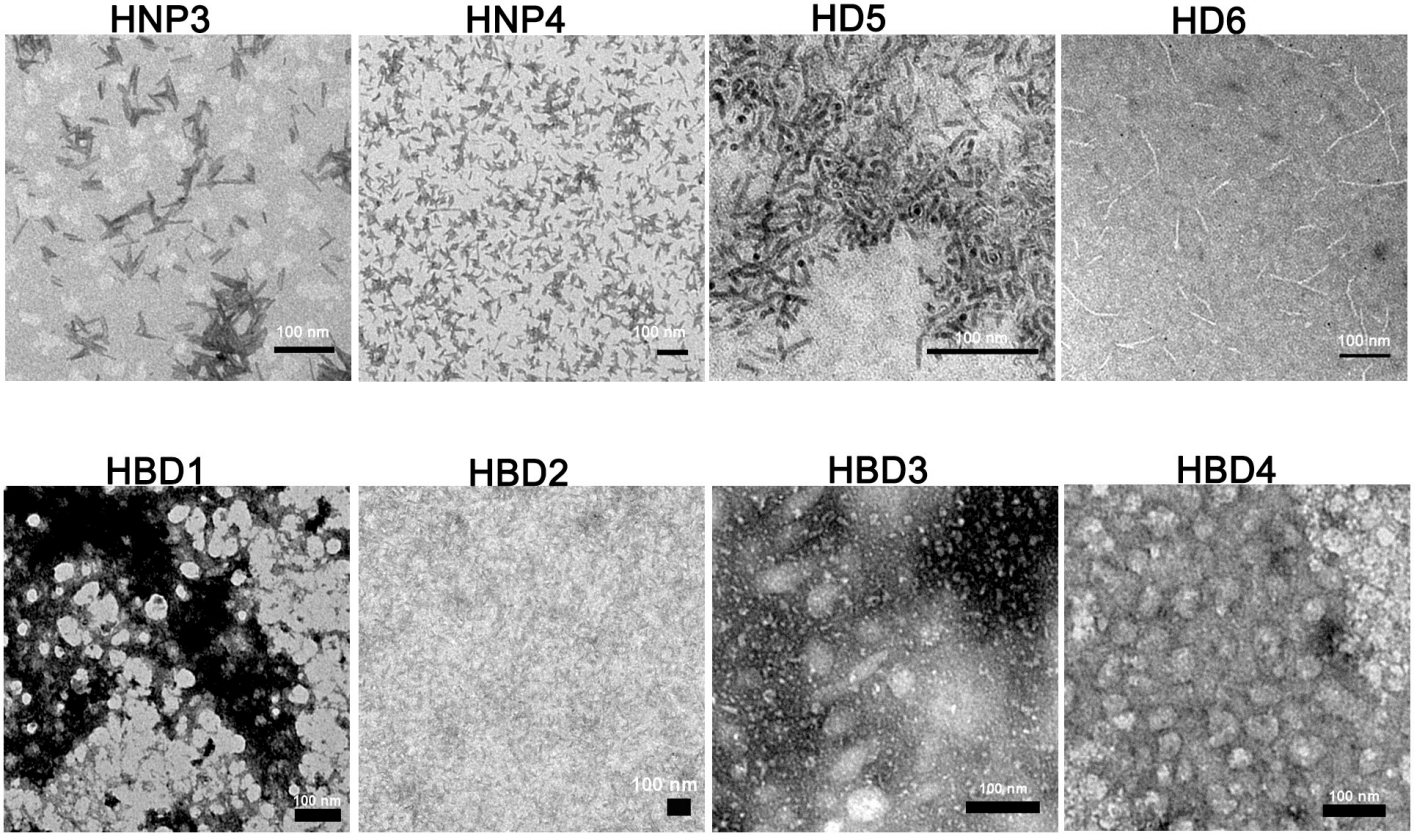
Transmission electron micrographs of human defensin aggregates.

The ability of human α-defensin HD6 to inhibit bacterial infection of epithelial cells has been attributed to its ability to self-assemble and form fibrillar mesh-like structures [53, 63]. Since other human defensins have also been reported to form higher order oligomers under crystalline state and in solutions [10–12, 15, 64], we compared the self-assembling properties of α- and β-defensins. Electron micrographs of human defensins are shown in Fig 7. While distinctive fibrillar morphology is observed for HD6 similar structures are not observed for other defensins. HNP3 and HD5 show rod-like structures while others show amorphous structures. Considering the fact that HD6’s ability to form fibrils *in vitro* is critical to its ability to form “nanonets” on binding to bacterial surfaces [53, 63], it is presumable that rod-like aggregates formed by HD5 and HNP3 will also likely to play a deciding role in their antibacterial activity and bacterial killing mechanism. Even subtle changes in the dimer interface, which is likely to modulate self-assembling properties, has been reported to affect the antibacterial potency and selectivity of HNP1 and HD5 [65, 66]. The physico-chemical properties of self-assembled aggregates of defensins on bacterial surfaces might be playing critical roles in their ability to interact with bacterial surface and membranes.

## Conclusions

In this study, we have compared various aspects of bacterial and model membrane permeabilizing abilities of human defensins. There are considerable variations in their ability to interact with the *E*. *coli* cell surface and model membranes, suggesting differences in the mechanisms by which human defensins exert their antibacterial activity. Although the arguments presented in this paper are based on the observations on *E*. *coli*, similar variations in the mechanism of bacterial killing can be anticipated for other gram-negative species as well. Since all the defensins described in this study are not active against *S*. *aureus*, our investigations have been confined to *E*. *coli*. Defensins show differences in membrane destabilization which could result in variations in bacterial killing mechanisms despite having very similar three dimensional structures. It is evident that the topography of positively selected amino acids during the evolution play a critical role in rendering highly heterogeneous mechanisms of bacterial killing without affecting their overall three dimensional fold.

## Supporting information

S1 Fig Accumulation of SYTOX green in *E*. *coli* treated with HD5 after 30 minutes of incubation. Arrows indicate the accumulation of SYTOX green. Numbers given in the upper left corner represent the elapsed time (h:min:s:ms).
(TIF)
