# Supplementary figures and images for: Variations in the interaction of human defensins with Escherichia coli: Possible implications in bacterial killing
Source: PLoS One. 2017 Apr 19;12(4):e0175858. doi: 10.1371/journal.pone.0175858 (PMC5397029; doi:10.1371/journal.pone.0175858)

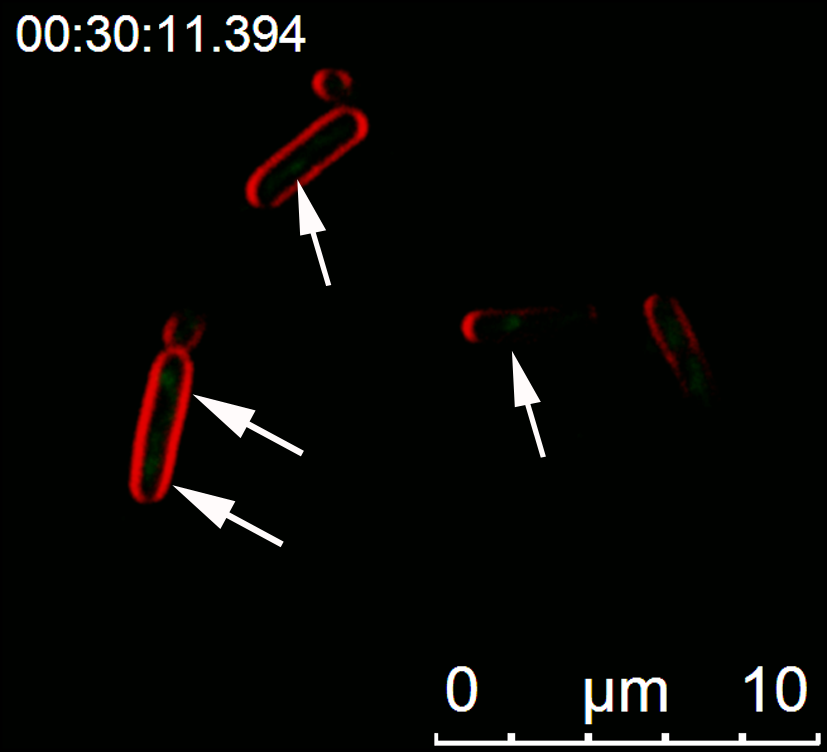

Supplement: S1 Fig — Arrows indicate the accumulation of SYTOX green. Numbers given in the upper left corner represent the elapsed time (h:min:s:ms). (TIF) [file pone.0175858.s001.tif]
